# Gene annotation bias impedes biomedical research

**DOI:** 10.1038/s41598-018-19333-x

**Published:** 2018-01-22

**Authors:** Winston A. Haynes, Aurelie Tomczak, Purvesh Khatri

**Affiliations:** 1 Stanford Institute for Immunity, Transplantation, and Infection, Stanford University, Stanford, California, USA; 2 Stanford Center for Biomedical Informatics Research, Department of Medicine, Stanford University, Stanford, California, USA; 3 Biomedical Informatics Training Program, Stanford University, Stanford, California, USA

## Abstract

We found tremendous inequality across gene and protein annotation resources. We observed that this bias leads biomedical researchers to focus on richly annotated genes instead of those with the strongest molecular data. We advocate that researchers reduce these biases by pursuing data-driven hypotheses.

## Introduction

After analyzing samples with a high throughput technology, the de facto first step is to perform pathway or network analysis to identify biological processes that are statistically enriched in the data^1^. Researchers typically form hypotheses for their follow up experiments based on the genes or proteins involved in the enriched processes. Commonly used resources for identifying gene functions and interactions include the Gene Ontology (GO)^2^, Reactome^3^, Comparative Toxicogenomics Database (CTD)^4^, DrugBank^5^, Protein Data Bank (PDB)^6^, Pubpular^7^, and NCBI GeneRIF. Since these resources are created by curation of the scientific literature, they typically only contain functional annotations for genes with published experimental data. Although GO includes predicted functional annotations for genes, they are considered of low quality^8^. Consequently, researchers select those genes or proteins for further validation that have prior experimental evidence, which, in turn, leads to more functional annotations for those genes at the expense of under-studied genes^9–12^.

We hypothesized that this experimental paradigm has led to a gene-centric disease research bias where hypotheses are confounded by the streetlight effect of looking for “answers where the light is better rather than where the truth is more likely to lie”^13–16^. To test this hypothesis, we examined the annotation inequality for the human genome across a number of biomedical databases using the Gini coefficient, which is a measure of inequality such that high coefficient value indicates higher inequality^17^.

## Results

### Annotation inequality is increasing over time

**Figure 1:**
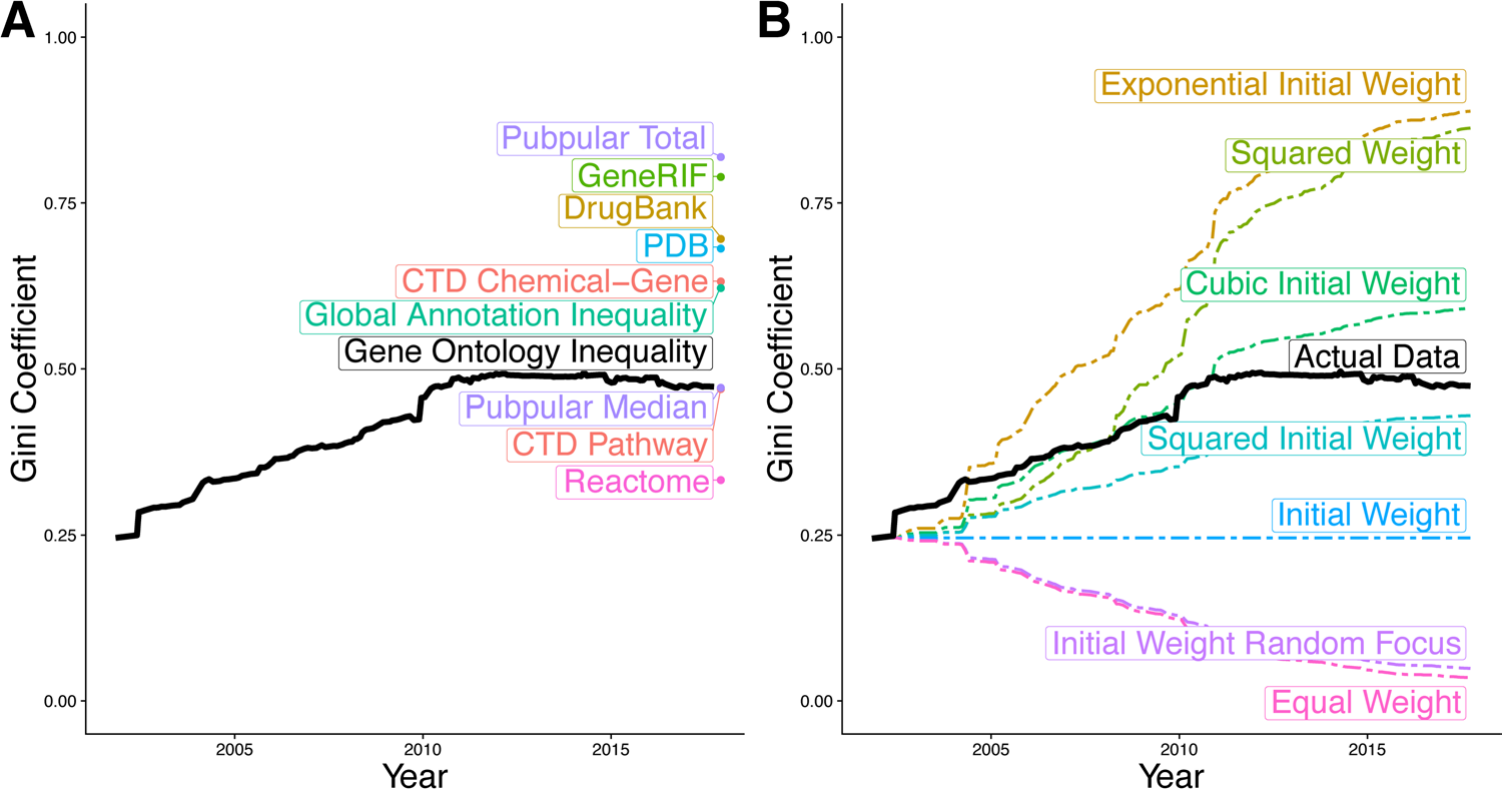
Inequality in gene annotations. (**A**) We measured the Gini coefficient across a variety of gene annotation resources. (**B**) We compared the growth in the Gini coefficient of the Gene Ontology to different models of increasing and decreasing inequality. See also Figure S1.

Despite the tremendous growth of Gene Ontology Annotations (GOA) from 32,259 annotations for 9,664 human genes in 2001 to 185,276 annotations for 17,314 genes in 2017, annotation inequality in GO has increased from a Gini coefficient of 0.25 in 2001 to 0.47 in 2017 (Fig. 1A) with tight confidence intervals (Figure S1A). We compared inequality in GOA data using eight inequality metrics: Gini coefficient, Ricci-Schutz coefficient, Atkinson’s measure, Kolm’s measure, Theil’s entropy, coefficient of variation, squared coefficient of variation, and generalized entropy. We observed increases in inequality over time irrespective of the metric used (Figure S1B). We used the Gini coefficient for the remainder of this manuscript since it demonstrated the most conservative estimate of the increase in inequality. Similarly, GOA inequality trends are not substantially affected by the inclusion or exclusion of particular types of annotations or ontology terms (Figure S1C).

We simulated changes in GOA equality using the first GO release as a baseline measurement. We estimated how inequality levels would have changed under different models, including equal growth across genes, growth consistent with the initial levels of inequality, and growth increasingly biased towards genes that began with many annotations. When we compared these different trajectories, we observed that the actual changes in inequality most closely matched the models of increasingly biased growth (Fig. 1B). Our findings further validate that genes with existing annotations continue to receive even more annotations^18^.

### Annotation inequality persists across organisms and databases

We computed annotation inequality in 12 other organisms over time for comparison including arabidopsis, chicken, cow, dicty, dog, fly, mouse, pig, rat, worm, yeast, and zebrafish (Figure S1A). When comparing the first version for each organism, human annotations exhibited the second greatest level of equality. In the most current versions of Gene Ontology annotations, humans exhibit the fourth highest inequality. The longitudinal trends varied across organisms, including organisms with both increasing and decreasing inequalities. Mouse and rat, the primary model organisms for human disease, exhibit increases in Gene Ontology annotations that are consistent with the patterns observed in the human data.

We examined other gene annotation databases to ensure that the observed phenomena was not specific to the GO. Other pathway databases, including Reactome (Gini = 0.33)^3^ and the CTD Pathway (Gini = 0.47)^4^, have a similarly high level of inequality (Fig. 1A). Indeed, every gene annotation resource we examined displayed a similarly high level of annotation inequality, including CTD chemical-gene associations (Gini = 0.63)^4^, PDB 3D protein structures (gini = 0.68)^6^, DrugBank drug-gene associations (Gini = 0.70)^5^, GeneRIF gene publication annotations (Gini = 0.79), and Pubpular disease-gene publication associations (Gini = 0.82)^7,19^. When considering the number of annotations pooled across all these databases, global gene annotation Gini coefficient was 0.63.

### Annotation inequality bias affects biomedical research

Next, we explored whether disease research may be affected by the inequality in gene annotation databases. Concerns that most published findings are false^20^, many results are inflated^21^, and research funding is being wasted^22,23^ have led to a number of proposals for reproducible and clinically relevant findings^24–26^. We have previously described a multi-cohort analysis framework^27–29^ that leverages biological and technical heterogeneity across multiple independent datasets to identify robust disease signatures. Using this framework, we have repeatedly demonstrated that it can identify robust disease signatures across a broad spectrum of diseases including organ transplant^27^, infections^30–33^, autoimmune disease^34^, cancer^35–37^, vaccination^38^, and neurodegenerative diseases^39^ for identifying diagnostic and prognostic markers, novel drug targets, and repurposing FDA-approved drugs.

**Figure 2:**
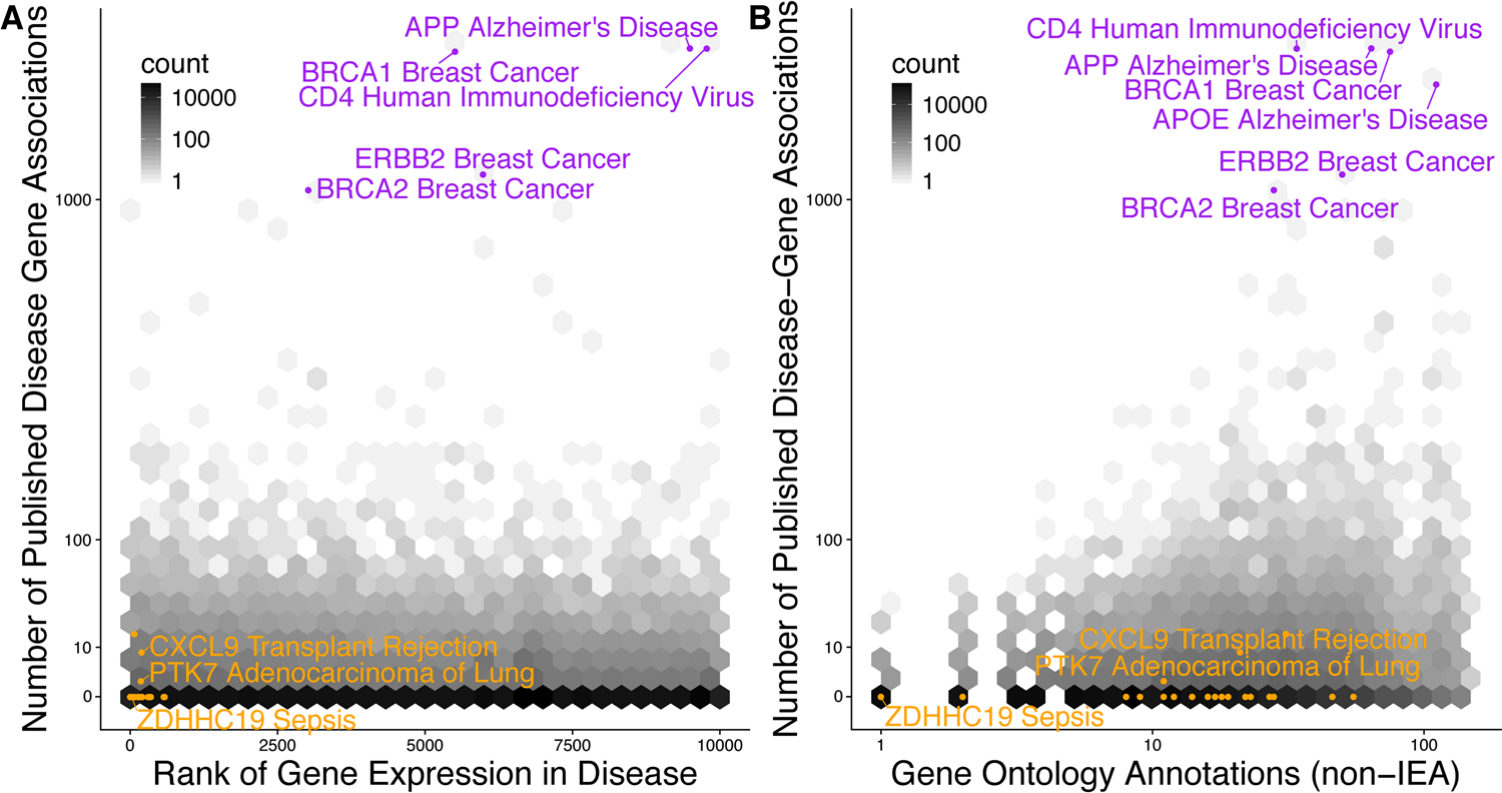
Published Disease-Gene Associations Not Reflected in Molecular Data. (**A**) The number of publications for every disease-gene pair was not significantly correlated with the gene expression multicohort analysis effect size FDR rank [Spearman’s correlation = −0.003, p = 0.836]. (**B**) The number of publications for every disease-gene pair correlated with the number of non-inferred from electronic annotation (non-IEA) Gene Ontology annotations [Spearman’s correlation = 0.110, p = 2.1e–16]. Orange points represent disease-gene associations published in our prior meta-analyses^27,30,37^. Purple points have at least 1000 publications. See also Figure S2.

In our manually curated meta-analyses of 104 distinct human conditions, we have integrated transcriptome data from over 41,000 patients and 619 studies to calculate an effect size for disease-gene associations^28^. Our analyses included diverse classes of human conditions such as cancer, autoimmune disease, viral infection, neurodegenerative and psychiatric disorders, pregnancy, and obesity. For these conditions, we extracted all disease gene associations with at least ten publications^7,19^. Published disease-gene associations exhibited no significant correlation with differential gene expression false discovery rate (FDR) rank (Spearman’s correlation = −0.003, p = 0.836, Fig. 2A) Overall, only 19.5% of published disease-gene associations were identified in gene expression analyses at a FDR of 5% that is consistent with previous publications that have successfully replicated between 11–25% of research studies^40,41^ (Figure S2A).

To observe whether this phenomenon was specific to gene expression, we extracted genome wide significant single nucleotide polymorphisms (SNPs) from the GWAS catalog^42^. We observed a non-significant correlation between the number of publications and SNP p-values, indicating a lack of concordance between genetic mutations and disease-gene publications (Spearman’s correlation = 0.017, p = 0.836, Figure S2B).

Based on these results, we hypothesized that the lack of correlation with molecular evidence may be an artifact of research bias towards well-characterized genes. Therefore, we examined correspondence between publications about a disease-gene pair and existing knowledge about that gene as indicated by the number of GO annotations. Indeed, the number of GO annotations for a gene of interest was significantly correlated with the published disease-gene associations (Spearman’s correlation = 0.110, p = 2.1e-16, Fig. 2B), but not with gene expression effect size FDR rank in disease (Spearman’s correlation = −0.023, p = 0.080, Figure S2C)^2^.

Many of the highly published disease-gene associations may have been studied for reasons that would not be directly reflected in gene expression analysis, including BRCA1 in breast cancer and CD4 in human immunodeficiency virus. The more troubling bias occurs when associations with strong molecular evidence have no publication record. Disease-gene associations we have reported in our published meta-analyses were typically novel findings with few Gene Ontology annotations, despite having extremely low false discovery rates and high effect sizes^27,30,35^ (orange points in Fig. 2). We observed similar patterns when we performed the same analysis on similar publication and GWAS data from HuGE Navigator^43,44^ (Figure S2D–F).

## Discussion

Collectively, our results provide an evidence of a strong research bias in literature that focuses on well-annotated genes instead of those with the most significant disease relationship in terms of both expression and genetic variation. We show that the inequality follows a “rich-getting-richer” pattern, where annotation growth is biased towards genes that were richly annotated in the initial versions of GO. We believe this stems from the typical experimental design. To illustrate this, consider an omics experiment that generates a list of hundreds or thousands of interesting genes. To interpret these genes, researchers use GO and pathway analysis tools. The researchers then generate targeted hypotheses for validation by interpreting the list of significant GO terms, focusing the genes or proteins annotated with that GO term. The researchers learn more about those targeted genes, leading to additional GO annotations for the already annotated genes. In this process, the list of unannotated genes is simply ignored because pathway analysis tools cannot map them to any GO terms. Hence, the self-perpetuating cycle of inequality continues.

While focusing research on the best characterized genes may be natural because it is easy to formulate a mechanistic hypothesis of the gene’s function in disease, we propose that the researchers in the era of omics should instead allow data to drive their hypotheses. We have repeatedly shown that expanding research outside of the streetlight of well characterized genes identifies novel disease-gene relationships^35–37^, identifies FDA-approved drugs that can be repurposed for other diseases^27^, and identifies clinically translatable diagnostic and prognostic disease signatures^27,30–34,39^. For example, we have previously identified *PTK7* as causally involved in non-small cell lung cancer^37^. At the time of publication, *PTK7* was labelled as an orphan tyrosine kinase receptor. In a very short span, this finding was transformed into an antibody-drug conjugate targeting *PTK7* that induced sustained tumor regression, outperformed standard-of-care chemotherapy, and reduced frequency of tumor-initiating cells in a preclinical study^45^. A Phase 1 clinical trial (NCT02222922) of *PTK7* antibody-drug conjugate, PF-06647020, has already completed with acceptable and manageable safety profile, and is now being considered for further clinical development. To enable researchers to pursue data-driven hypotheses, we have made our rigorously validated gene expression multicohort analysis data publicly available (http://metasignature.stanford.edu) where it may be explored based on either diseases or genes of interest^29,46^. Focusing on genes with the strongest molecular evidence instead of the most annotations would enable researchers to break the self-perpetuating annotation inequality cycle that results in research bias.

## Methods

### Inequality metrics calculations

We used the R package *ineq* to compute eight inequality metrics: (1) Gini coefficient, (2) Ricci-Schutz coefficient, (3) Atkinson’s measure, (4) Kolm’s measure, (5) Theil’s entropy, (6) coefficient of variation, (7) squared coefficient of variation, and (8) generalized entropy.

### Gini coefficient

The R package ineq^47^ calculates the Gini coefficient as:

1 $$G=\frac{\sum _{i=1}^{n}\sum _{j=1}^{n}|{x}_{i}-{x}_{j}|}{2n\sum _{i=1}^{n}{x}_{i}}$$

where *n* is the number of genes and *x*_*i*_ is the number of annotations for a gene *i*^17^. We included all human genes with at least one annotation in the Gini calculations.

### List of human gene names

We used the Entrez Gene list downloaded in February 2017 of 20,698 current, protein-coding, human genes as our source of human genes.

### Gene Ontology Annotations data

We calculated the number of annotations for each human gene in the Gene Ontology^2^. in every version of GO annotations since 2001 that was available at http://http.ebi.ac.uk/pub/databases/GO/goa/old. Duplicate annotations that only differ in evidence codes were counted once.

We examined the Gini coefficient for the different classes of evidence codes (experimental, computational analysis, author statement, curatorial statement, and automatically assigned) and namespaces (cellular component, biological process, molecular function). We found no substantial differences in the Gini coefficient values and trends regardless of the terms being considered (Figure S1D). To focus on terms with the strongest evidence, the remainder of our manuscript excluded the evidence codes IEA, ND, and NR^8^. To focus on terms related to functional understanding of genes, we only considered the biological process and molecular function GO namespaces.

### GOA for other organisms

We downloaded historic Gene Ontology annotation data for all 12 organisms available from http://http.ebi.ac.uk/pub/databases/GO/goa/old/. These organisms included arabidopsis, chicken, cow, dicty, dog, fly, mouse, pig, rat, worm, yeast, and zebrafish.

### Confidence intervals

Using bootstrap resampling, we calculated 95% confidence intervals around our Gini coefficients based on 1000 permutations of each version of the human Gene Ontology annotation data [Figure S1B].

### Modeling Gini coefficient over time

We used the first available version of the human GO annotations (http://http.ebi.ac.uk/pub/databases/GO/goa/old/HUMAN/gene_association.goa_human.1.gz) as our baseline measurement in all models. We modeled every release of GO under different growth models, distributing the number of new annotations from that release across genes according to the model. We define our update step as:

$${n}_{{{\rm{gene}}}_{{i}_{j+1}}}={n}_{{{\rm{gene}}}_{{i}_{j}}}+{n}_{j+1}\ast {p}_{{{\rm{gene}}}_{i}}$$

where:

${n}_{{{\rm{gene}}}_{{i}_{j}}}$ is the number of annotations for gene_*i*_ at timestep *j*

*n*_*j*_ _+_ _1_ is the number of annotations added in version *j* + 1 (subject to *n* ≥ 0).

${p}_{{{\rm{gene}}}_{i}}$ be the probability of annotation being assigned to gene_*i*_

${p}_{{{\rm{gene}}}_{{i}_{0}}}$ be the initial proportion of annotations assigned to gene_*i*_ in the initial release of GO $(\frac{{n}_{{{\rm{gene}}}_{{i}_{0}}}}{{n}_{0}})$.

For each model, we define our ${p}_{{{\rm{gene}}}_{i}}$ as follows:

**Exponential initial weight**. ${p}_{{{\rm{gene}}}_{{\rm{i}}}}={\exp }^{{p}_{{{\rm{gene}}}_{{i}_{0}}}}$. Models inequality growth consistent with exponential initial probability.

**Cubic initial weight**. ${p}_{{{\rm{gene}}}_{i}}={({p}_{{{\rm{gene}}}_{{i}_{0}}})}^{3}$. Models inequality growth consistent with cubic initial probability.

**Squared weight**. ${p}_{{{\rm{gene}}}_{i}}={({p}_{{{\rm{gene}}}_{i-1}})}^{2}$. Models inequality growth consistent with squared probability from previous round.

**Squared initial weight**. ${p}_{{{\rm{gene}}}_{i}}={({p}_{{{\rm{gene}}}_{{i}_{0}}})}^{2}$. Models inequality growth consistent with squared initial probability.

**Initial weight**. ${p}_{{{\rm{gene}}}_{i}}={p}_{{{\rm{gene}}}_{{i}_{0}}}$. Models inequality growth consistent with initial probability.

**Initial weight random focus**. ${p}_{{{\rm{gene}}}_{i}}={p}_{{\rm{random}}{{\rm{gene}}}_{{i}_{{\rm{0}}}}}$ where ${p}_{{\rm{random}}{{\rm{gene}}}_{{i}_{{\rm{0}}}}}$ is the initial probability from a randomly selected gene. Model assumes inequal growth in annotations consistent with the initial probabilities but randomized across genes in every version of GO.

**Equal weight**. ${p}_{{{\rm{gene}}}_{i}}=\frac{{n}_{j+1}}{|{\rm{genes}}|}$ where |genes| is the number of genes in GO. Models even growth of annotations across genes.

### Other gene annotation database Gini coefficient calculation

**Pubpublar**. We manually downloaded gene-publication data in August 2016 from Pubpular for 102 of the diseases in our gene expression database^7,19^. “Pubpular Total” refers to the inequality of gene-publication data across all diseases. “Pubpular Median” refers to the median inequality of gene-publication for each disease.

**Reactome**. We downloaded Reactome pathway data from the complete database release 59^3^. We downloaded data in MySQL format and parsed pathways into UniProt identifiers using custom scripts. We converted UniProt identifiers to gene names using the UniProt identifier conversion tool^48^. We calculated the number of pathways including each gene name.

**CTD**. We downloaded the CTD^4^ data in February 2017, with the chemical-gene associations and the gene-pathway associations. We calculated the number of chemical-gene and gene-pathway associations for each gene name.

**GeneRIFs**. We downloaded GeneRIFs from the NCBI in February 2017. We included all human GeneRIFS (Tax ID: 9606). We calculated the number of GeneRIFs for each gene.

**Protein Data Bank**. We downloaded the gene names associated with protein structures from the Protein Data Bank^6^ in February 2017 and calculated the number of structures per gene name.

**DrugBank**. We downloaded the DrugBank^5^ database version 5.0.5 and identified all drugs with known activities on human genes. We calculated the number of drugs targeting each gene.

### Gene expression data collection and multicohort analysis

Gene expression multicohort analysis data was compiled from the MetaSignature database^28^. MetaSignature includes data from manual multicohort analysis of over 41,000 samples, 619 studies, and 104 diseases. Briefly, relevant data were downloaded from Gene Expression Omnibus and ArrayExpress^49,50^. Cases and controls were manually labeled for each disease and multicohort analysis was performed using the MetaIntegrator package^28^. We used the Hedges’ *g* summary effect size, standard error, and false discovery rate which the MetaIntegrator package calculates for every gene.

### Data collection for disease-gene publications and SNP data

We downloaded the number of publications for each disease-gene relationship from PubPular and HuGE Navigator in August 2016 for as many of the 104 disease in MetaSignature as were present in the databases (102 in PubPular and 81 in HuGE)^7,19,43^. PubPular gave the top 261 gene associations, and HuGE gave all known associations. For all correlations, we only considered disease-gene associations with at least 10 publications to limit false positive associations.

We downloaded disease-SNP relationships, including gene mappings, odds ratios, and p-values, from the GWAS Catalog and HuGE Navigator for 61 and 54, respectively, of the 103 diseases in MetaSignature^42,44^. From Gene Ontology, we calculated the counts of non-Inferred from Electronic Annotation annotations for all the genes in the MetaSignature database^2^. The Spearman rank correlation was used for all correlations.

Our plots show the top 10,000 gene associations for each disease by effect size FDR rank. Correlation calculations do not include a similar limit.

### Code and data availability

The code and data we used to run this analysis is available at https://khatrilab.stanford.edu/researchbias and https://figshare.com/projects/Gene_annotation_bias_impedes_biomedical_research/27124 (10.6084/m9.figshare.5660824.v2 and 10.6084/m9.figshare.5648332.v6).

## Electronic supplementary material

Supplemental Figures
