# Supplementary material for: Gene annotation bias impedes biomedical research
Source: Sci Rep. 2018 Jan 22;8:1362. doi: 10.1038/s41598-018-19333-x (PMC5778030; doi:10.1038/s41598-018-19333-x)
Supplement: Supplementary file 1 — Supplemental Figures [file 41598_2018_19333_MOESM1_ESM.pdf]

Supplemental Information: Gene annotation bias impedes biomedical research

Winston A. Haynes[1,2,3], Aurelie Tomczak[1,2], and Purvesh Khatri[1,2,*]

[1]Stanford Institute for Immunity, Transplantation, and Infection,Stanford University, Stanford, California, USA
[2]Stanford Center for Biomedical Informatics Research, Department of Medicine, Stanford University, Stanford, California, USA
[3]Biomedical Informatics Training Program,Stanford University, Stanford, California, USA
[*]Correspondence to pkhatri@stanford.edu

ABSTRACT

We found tremendous inequality across gene and protein annotation resources. We observe that this bias leads biomedical researchers to focus on richly annotated genes instead of those with the strongest molecular data. We advocate for researchers to reduce these biases by pursuing data-driven hypotheses.

Supplementary Materials

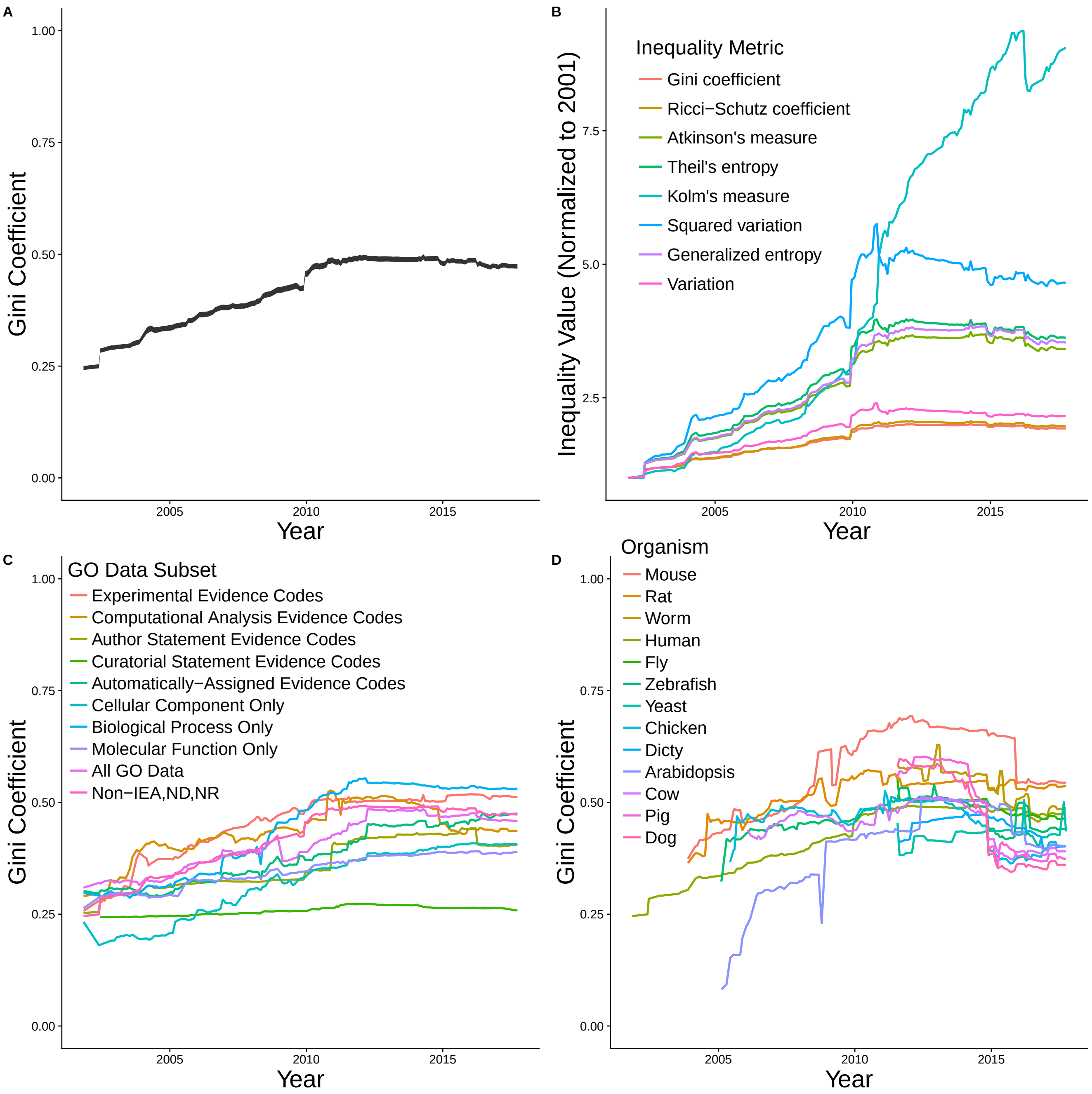

Figure S1. Related to Figure 1. (a) Confidence intervals for the human GO Gini coefficient based on 1000 bootstrap resamplings. (b) Comparison of different inequality metrics based on the human GO data. (c) Gini coefficient measured with different subsets of human GO data. (d) Gini coefficient measured across different organisms over time.

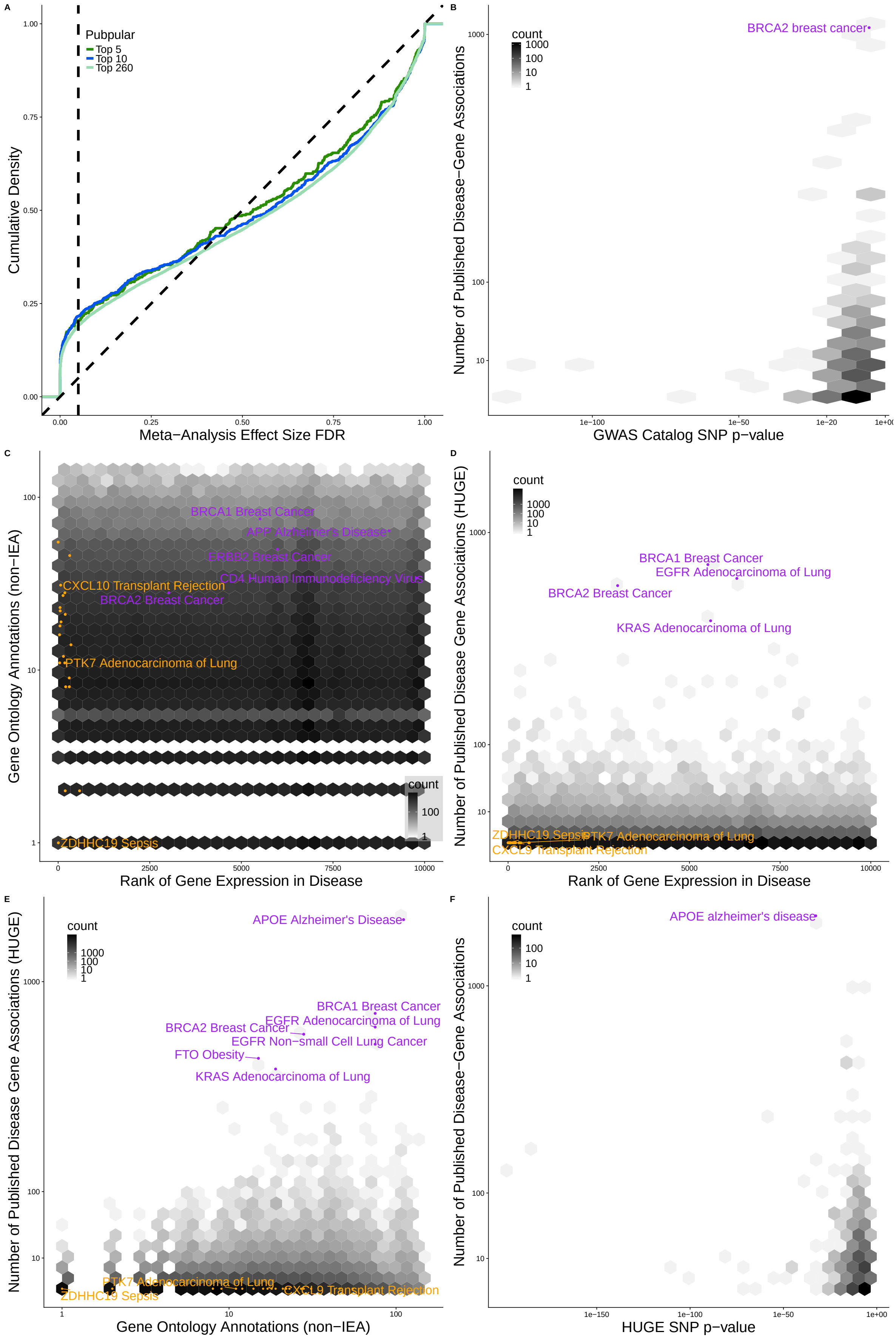
A
Pubpular
Top 5
Top 10
Top 260
Cumulative Density
Meta−Analysis Effect Size FDR
B
count
BRCA2 breast cancer
Number of Published Disease−Gene Associations
GWAS Catalog SNP p−value
C
BRCA1 Breast Cancer
APP Alzheimer's Disease
ERBB2 Breast Cancer
CD4 Human Immunodeficiency Virus
CXCL10 Transplant Rejection
BRCA2 Breast Cancer
PTK7 Adenocarcinoma of Lung
ZDHHC19 Sepsis
Gene Ontology Annotations (non−IEA)
Rank of Gene Expression in Disease
D
BRCA1 Breast Cancer
EGFR Adenocarcinoma of Lung
BRCA2 Breast Cancer
KRAS Adenocarcinoma of Lung
ZDHHC19 Sepsis
PTK7 Adenocarcinoma of Lung
CXCL9 Transplant Rejection
Number of Published Disease Gene Associations (HUGE)
Rank of Gene Expression in Disease
E
APOE Alzheimer's Disease
BRCA1 Breast Cancer
EGFR Adenocarcinoma of Lung
BRCA2 Breast Cancer
EGFR Non−small Cell Lung Cancer
FTO Obesity
KRAS Adenocarcinoma of Lung
PTK7 Adenocarcinoma of Lung
ZDHHC19 Sepsis
CXCL9 Transplant Rejection
Number of Published Disease Gene Associations (HUGE)
Gene Ontology Annotations (non−IEA)
F
APOE alzheimer's disease
Number of Published Disease−Gene Associations
HUGE SNP p−value

Figure S1. Related to Figure 2. (a) Only 19% of published disease-gene associations have gene effect size FDR of less than 5%. Cumulative distributions of the top 5, 10, and 250 disease-gene associations for each disease from PubPular database. Vertical line at a gene expression meta-analysis effect size FDR of 5%. (b) The number of publications for every disease-gene pair is not significantly correlated with published results from SNP GWAS from the GWAS catalog [Spearman's correlation = 0.017, p = 0.836]. (c) The number of gene ontology annotations for every gene is not correlated with the gene expression meta-analysis effect size false discovery rate (FDR) rank [Spearman's correlation = -0.010, p = 0.156]. (d) The number of publications for every disease-gene pair vs. the gene expression meta-analysis effect size FDR rank based on the HuGE Navigator data [Spearman's correlation = 0.030, p = 0.170]. (e) The number of publications for every disease-gene pair vs. the number of non-inferred from electronic annotation (non-IEA) Gene Ontology annotations based on the HuGE Navigator data [Spearman's correlation = 0.121, p = 5.5e-8]. (f) The number of publications for every disease-gene pair vs. SNP GWAS p-value based on the HuGE Navigator data [Spearman's correlation = -0.204, p = 1.4e-3].
